# Low thrombin generation predicts poor prognosis in ischemic stroke patients after thrombolysis

**DOI:** 10.1371/journal.pone.0180477

**Published:** 2017-07-10

**Authors:** Renáta Hudák, Edina G. Székely, Katalin R. Kovács, Attila Nagy, Gergely Hofgárt, Ervin Berényi, László Csiba, János Kappelmayer, Zsuzsa Bagoly

**Affiliations:** 1 Department of Laboratory Medicine, Faculty of Medicine, University of Debrecen, Debrecen, Hungary; 2 Department of Laboratory Medicine, Division of Clinical Laboratory Sciences, Faculty of Medicine, University of Debrecen, Debrecen, Hungary; 3 Department of Neurology, Faculty of Medicine, University of Debrecen, Debrecen, Hungary; 4 Department of Preventive Medicine, Faculty of Public Health, University of Debrecen, Debrecen, Hungary; 5 Department of Radiology, Faculty of Medicine, University of Debrecen, Debrecen, Hungary; University of Modena and Reggio Emilia, ITALY

## Abstract

Thrombolysis by intravenous recombinant tissue plasminogen activator (rt-PA) is an effective therapy in acute ischemic stroke (AIS). Thrombin generation test (TGT) is a global hemostasis test providing information about the speed and amount of generated thrombin in plasma. Here we aimed to find out whether results of this test before the initiation of thrombolysis might predict outcomes. Study population included 120 consecutive AIS patients, all within 4.5 hours of their symptom onset, who underwent thrombolysis by rt-PA. Blood samples were collected from all patients upon admission and TGT was performed using platelet poor plasma. Clinical data of patients including the NIHSS were registered at admission, day 1 and 7 after therapy. The ASPECT score was assessed using CT images taken before and 24 hours after thrombolysis. Long-term functional outcome was defined 3 months after the event by the modified Rankin Scale. Endogenous Thrombin Potential (ETP) and Peak Thrombin were significantly lower in patients with cardioembolic IS. Symptomatic intracranial hemorrhage (SICH) was found in 6 patients and was significantly associated with low ETP and Peak Thrombin levels. A multiple logistic regression model revealed that an ETP result in the lower quartile is an independent predictor of mortality within the first two weeks (OR: 6.03; 95%CI: 1.2–30.16, p<0.05) and three months after the event (OR: 5.28; 95%CI: 1.27–21.86, p<0.05). Low levels of ETP and Peak Thrombin parameters increase the risk of therapy associated SICH. A low ETP result is an independent predictor of short- and long-term mortality following thrombolysis.

## Introduction

Early intravenous administration of recombinant tissue plasminogen activator (rt-PA) has been proven to be an effective therapy for acute ischemic stroke (AIS) [1,2]. Although those eligible for this therapy are carefully selected, about 6% of patients undergoing thrombolysis develop potentially fatal intracranial hemorrhage as a side effect [3]. On the other hand, in a subset of patients thrombolysis is inefficient and due to the failure of recanalization no clinical improvement is observed [4]. Today, these events cannot be foreseen at the initiation of the therapy and only few conventional risk factors (e.g. age, stroke severity, hyperglycemia, etc.) may be considered to predict outcomes. In theory, the lysis of the clot is likely to depend on factors influencing clot structure. Given the complexity of the hemostasis system, which involves several interrelated procoagulant and anticoagulant pathways, measuring the levels of individual proteins might have limited utility in the prediction of therapeutic outcomes. Thrombin generation test (TGT) is a global hemostasis test providing information about the speed and amount of generated thrombin in plasma [5]. Here we aimed to find out whether results of this test before the initiation of therapy might predict outcomes after thrombolysis.

## Materials and methods

### Study population

Patients were enrolled between March 2011 and January 2013 in a single Stroke Center (Department of Neurology, Faculty of Medicine, University of Debrecen, Debrecen, Hungary). Study population included consecutive acute ischemic stroke patients admitted within 4.5 hours of their symptom onset undergoing intravenous (i.v.) thrombolytic therapy according to the European Stroke Organization guidelines [6] using recombinant tissue plasminogen activator (rt-PA, Alteplase, Boehringer Ingelheim, Germany). Patients who were on anticoagulant therapy at admission were excluded from the study population. All enrolled patients or their relatives had been informed about the study and gave written informed consent. The study was approved by the Ethics Committee of University of Debrecen, Debrecen, Hungary.

### Blood sampling and routine laboratory measurements

Peripheral blood samples were drawn from patients on hospital admission into vacutainer tubes (tubes with no anticoagulant for routine clinical chemistry tests, tubes anticoagulated with K_3_-EDTA for complete blood count and tubes containing 0.105 M sodium citrate for routine hemostasis tests, Becton Dickinson, Franklin Lakes, NJ). Plasma from tubes containing sodium citrate, theophylline, adenosine and dipyridamole (Vacuette CTAD Tubes, Greiner Bio-One, Vienna, Austria) was separated by centrifugation at 1220 g for 15 min, room temperature and samples were stored at -70°C until the determination of thrombin generation. Serum ions, glucose levels, basic kidney function tests, liver function tests, lipid profile and high sensitivity C-reactive protein (CRP) were determined by conventional methods (Roche Diagnostics, USA). Routine hemostasis tests were measured immediately after plasma separation (Siemens Healthcare Diagnostics, Marburg, Germany).

### Thrombin generation test

Thrombin generation test was performed as described previously using the Thrombinoscope CAT (Calibrated Automated Thrombogram, Maastricht, The Netherlands) assay according to the manufacturer’s instructions (Diagnostica Stago, Asnières, France) [7,8]. Briefly, 80 microliters of plasma was incubated with 20 μL PPP-Reagent^™^ (containing 5 pM recombinant tissue factor and 4 μM phospholipids) for 10 minutes in round-bottomed 96-well black microplates. For each sample, a calibrator (Thrombin Calibrator^™^) was run in parallel in order to correct the fluorescence signal for substrate consumption and plasma color variability. Thrombin generation was initiated by the addition of 20 μL of FluCa-Kit^™^ (a mixture of Fluorogenic substrate and Fluo-Buffer containing CaCl_2_). All samples were run in duplicates. Fluorescence was detected by a Fluoroskan Ascent^®^ fluorimeter (Thermo Fischer Scientific, Waltham, MA) and the thrombin generation curves were analysed by the Thrombinoscope software (Thrombinoscope BV, Maastricht, The Netherlands). Thrombin generation curves were characterised by the following parameters (calculated and presented by the Thrombinoscope software): 1/Lagtime: the moment at which thrombin generation starts, 2/Endogenous Thrombin Potential (ETP): the area under the curve, 3/Peak Thrombin: the highest thrombin concentration, 4/Time to Peak: the time until the Peak Thrombin, 5/Start Tail: the time to end-point of thrombin generation and 6/Velocity Index: the slope of the curve between the beginning of thrombin generation and the Time to Peak parameter.

### Clinical data

For each patient the time of symptom onset, demographic and clinical characteristics (smoking status, previous medication, neurological status) were recorded upon admission. Neurological deficit of patients was determined by the National Institute of Health Stroke Scale (NIHSS) [9] on admission (before thrombolysis) and at 2 h, 24 h and 7 days after thrombolytic therapy. Unfavourable short-term functional outcome was defined as an increase in the NIHSS by at least 4 points by day 7. Upon admission, all patients underwent computer tomography brain scan (CT) and CT angiography (CTA) to ensure the diagnosis of acute ischemic stroke. Stroke etiology was classified according to the Trial of Org 10172 in Acute Stroke Treatment (TOAST) criteria [10]. A control CT was performed for every patient 24 hours after rt-PA infusion. Hemorrhagic events on the control CT scan were classified as symptomatic (SICH) or asymptomatic intracranial hemorrhage (aSICH) according to the European Cooperative Acute Stroke Study (ECASS) II criteria [11]. CT images taken before and 24 hours after the therapy were analysed by 4 different investigators and the Alberta Stroke Program Early CT Scores (ASPECTS) were calculated [12]. Each patient was evaluated for the following cardiovascular risk factors: arterial hypertension, atrial fibrillation, hyperlipidemia and diabetes mellitus based on the use of medications or previous history. Patients were followed-up and long-term functional outcomes were determined at 3 months after the stroke event using the modified Rankin Scale (mRS). Unfavourable long-term outcome was defined as a mRS score greater than 1.

### Statistical analysis

Evaluation of the results was performed according to: stroke etiology, stroke severity (classification based on the baseline NIHSS: 0–5, 6–10, 11–15, >15), the presence of intracranial hemorrhage after thrombolysis treatment, short-term and long-term functional outcomes and mortality.

Normality of the data was evaluated by the Shapiro-Wilk test. ANOVA using the Bonferroni correction or Kruskal-Wallis test using the Dunn’s post test were applied for multiple comparisons. In all two-group analyses unpaired t-test or in case of non-parametric data Mann-Whitney U test was used. Pearson’s or Spearman’s correlation coefficient was used to determine the strength of correlation between parameters of the thrombin generation test and other continuous variables. A backward multiple logistic regression model was used to determine factors that are independent predictors of hemorrhagic transformation or death after thrombolysis. Variables were selected for entry into the multivariate model based on the results of univariate and correlation analyses, previous literature and other methodological principles (dichotomized variables wherever possible). Results of the logistic regression analysis were expressed as odds ratio (OR) and 95% confidence interval (CI). A p value of <0.05 was considered statistically significant. Statistical analysis was performed using Stata 12 (Stata Corp LP, Texas, USA) and GraphPad Prism 5.0 (GraphPad Prism Inc., La Jolla, CA, USA) softwares.

## Results

A total of 120 consecutive AIS patients receiving i.v. thrombolysis were included in the study. Baseline demographic characteristics of patients and stroke characteristics are shown in Tables 1 and 2. In case of 6 patients i.v. thrombolytic therapy was supplemented with intraarterial thrombolysis according to the standard protocol; the final dose of rtPA and the duration of thrombolysis was not significantly different in case of these patients. Median age was 69.0 (IQR: 59.0–79.0) years, 59.2% were men. Median NIHSS on admission was 8 (IQR: 5–14). Median time-to-treatment with rt-PA was 159 min (IQR: 125–180 min). According to the TOAST criteria, a major fraction of the patients had atherothrombotic stroke (large-artery atherosclerosis: 38.3%, small-vessel occlusion: 11.7%). A favourable short-term outcome was observed in 40% of the patients. Unfavourable short-term and long-term outcome was found in 14.2% and 46.7% of all cases, respectively. Mortality by day 7, day 14 and by the end of the 3^rd^ month was 4.2%, 12.5% and 21.7%, respectively. Mortality rates were not different according to the etiology of the stroke (data not shown). Hemorrhagic complications were detected in case of 13 patients, among which 6 had SICH (5% of total patient population). One patient had died due to intracranial hemorrhagic complication on the 6^th^ day of therapy. The occurrence of hemorrhagic complications was not significantly different in strokes of atherothrombotic origin as compared to cardioembolic strokes.

**Table 1:** Baseline characteristics of patients.

Characteristic	Values
n	120
Age (years), median (IQR)	69.0 (59.0–79.0)
Male, n (%)	71 (59.2)
Cerebrovascular risk factors, n (%)	
Arterial hypertension	91 (76.0)
Atrial fibrillation	28 (23.3)
Hyperlipidaemia	75 (62.5)
Diabetes mellitus	36 (30.0)
Previous stroke	37 (30.8)
Smoking, n (%)	
Non-smoker	63 (52.5)
Previous smoker	15 (12.5)
Active smoker	30 (25.0)
Undetermined	12 (10.0)
Serum glucose (mmol/L), median (IQR)	6.4 (5.5–7.8)
C-reactive protein (mg/L), median (IQR)	3.1 (1.5–5.7)
INR, median (IQR)	1.0 (0.9–1.0)
Current drug use, n (%)	
Antihypertensive therapy	84 (70.0)
Antiplatelet drug*	52 (43.3)
Lipid-lowering therapy	34 (28.3)
Undetermined	8 (6.7)
Thrombin generation parameters, median (IQR)	
Lagtime (min)	3.7 (3.0–4.2)
ETP (nM x min)	1482.0 (1266.0–1785.0)
Peak Thrombin (nM)	258.0 (204.7–315.3)
Time to Peak (min)	7.2 (6.3–8.4)
StartTail (min)	23.7 (21.8–25.4)
Velocity Index (nM/min)	73.3 (50.2–101.9)

IQR: interquartile range, INR: international normalized ratio, ETP: Endogenous Thrombin Potential.

*Aspirin or P2Y_12_ inhibitor treatment or both.

**Table 2:** Stroke characteristics.

Characteristic	Values
Time to treatment (min) with rt-PA, median (IQR)	159.0 (125.0–180.0)
Duration of thrombolysis treatment (min), median (IQR)	60.0 (60.0–64.0)
Dose of rt-PA, (mg±SD)	
Intravenous rt-PA	67.6±15.5
Bridging (combined intravenous and intraarterial)	68.0±12.5
Stroke etiology (TOAST), n (%)	
Large-artery atherosclerosis	46 (38.3)
Small-vessel occlusion	14 (11.7)
Cardioembolic	23 (19.2)
Other/undetermined	37 (30.8)
Stroke severity on admission, n (%)	
NIHSS 0–5	35 (29.2)
NIHSS 6–10	44 (36.7)
NIHSS 11–15	18 (15.0)
NIHSS >15	21 (17.5)
Undetermined	2 (1.6)
Short-term functional outcome, n (%)	
Favourable	48 (40.0)
No change	43 (35.8)
Unfavourable	17 (14.2)
Death	5 (4.2)
Undetermined	7 (5.8)
Haemorrhagic transformation (ECASS II), n (%)	13 (10.8)
aSICH	7 (5.8)
SICH	6 (5.0)
Mortality by day 7, n (%)	
Survival	115 (95.8)
Death	5 (4.2)
Mortality by day 14, n (%)	
Survival	105 (87.5)
Death	15 (12.5)
Long-term functional outcome at 3months, n (%)	
mRS 0–1	41 (34.2)
mRS 2–5	30 (25.0)
mRS 6 (death)	26 (21.7)
Undetermined	23 (19.1)
Imaging data (median, total range)	
ASPECTS on admission	10.0 (7.0–10.0)
ASPECTS at 24 h	9.0 (0.0–10.0)

rt-PA: recombinant tissue type plasminogen activator, TOAST: Trial of Org 10172 in

Acute Stroke Treatment, NIHSS: National Institute of Health Stroke Scale, ECASS II: European Cooperative Acute Stroke Study II, aSICH: asymptomatic intracranial hemorrhage, SICH: symptomatic intracranial hemorrhage, mRS: modified Rankin Scale, ASPECTS: Alberta Stroke Program Early CT Score.

### Thrombin generation as a predictor of thrombolysis outcome

**Fig 1:**
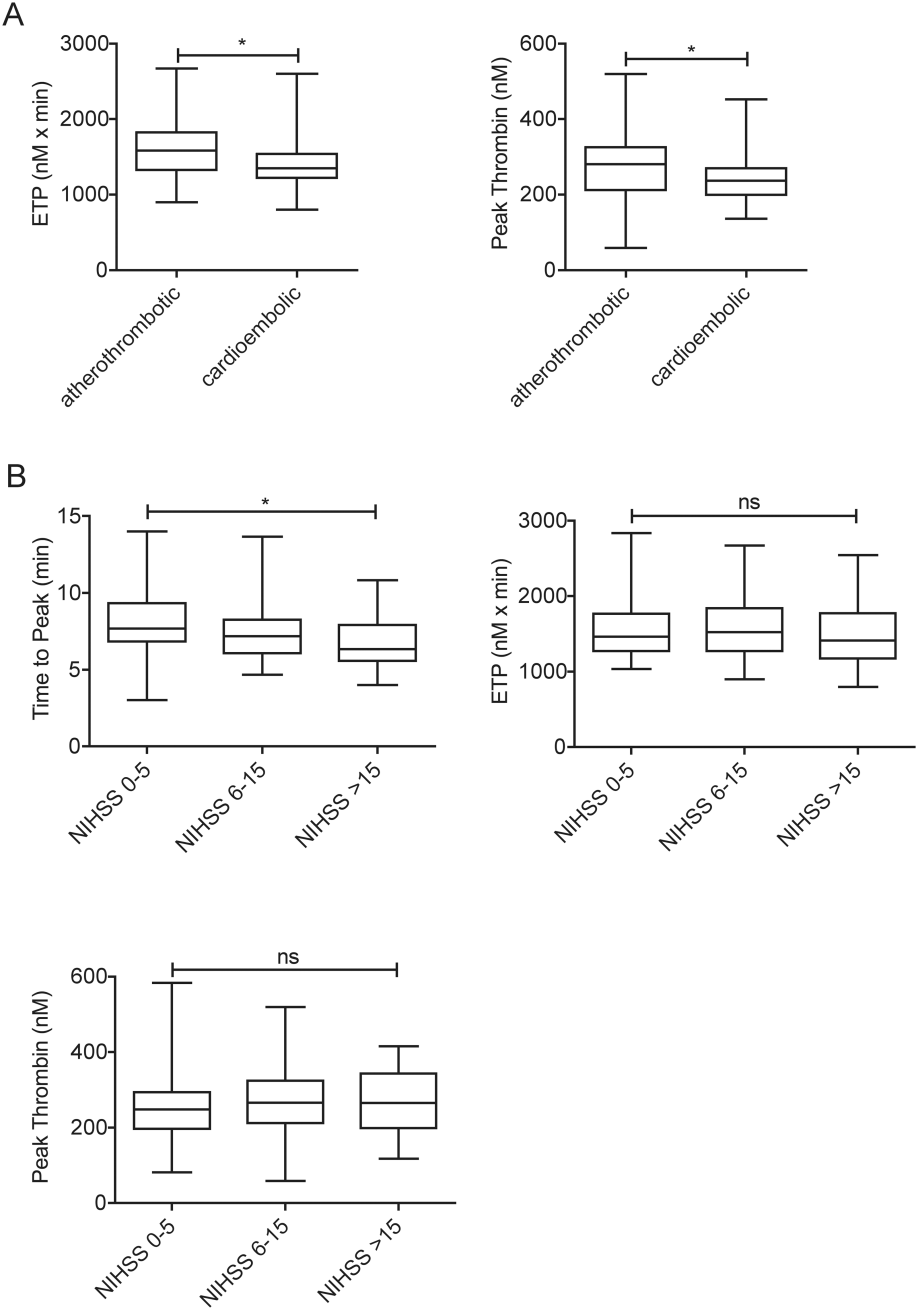
The association of thrombin generation parameters with the etiology (A) and the severity (B) of stroke. Box and whisker plots indicate median, interquartile range and total range. Statistical significance was assessed by Mann-Whitney U test (A) and Kruskal-Wallis and Dunn’s Multiple Comparison Test (B). *Statistically significant (p<0.05), ns: non-significant. ETP: Endogenous Thrombin Potential, NIHSS: National Institutes of Health Stroke Scale.

**Fig 2:**
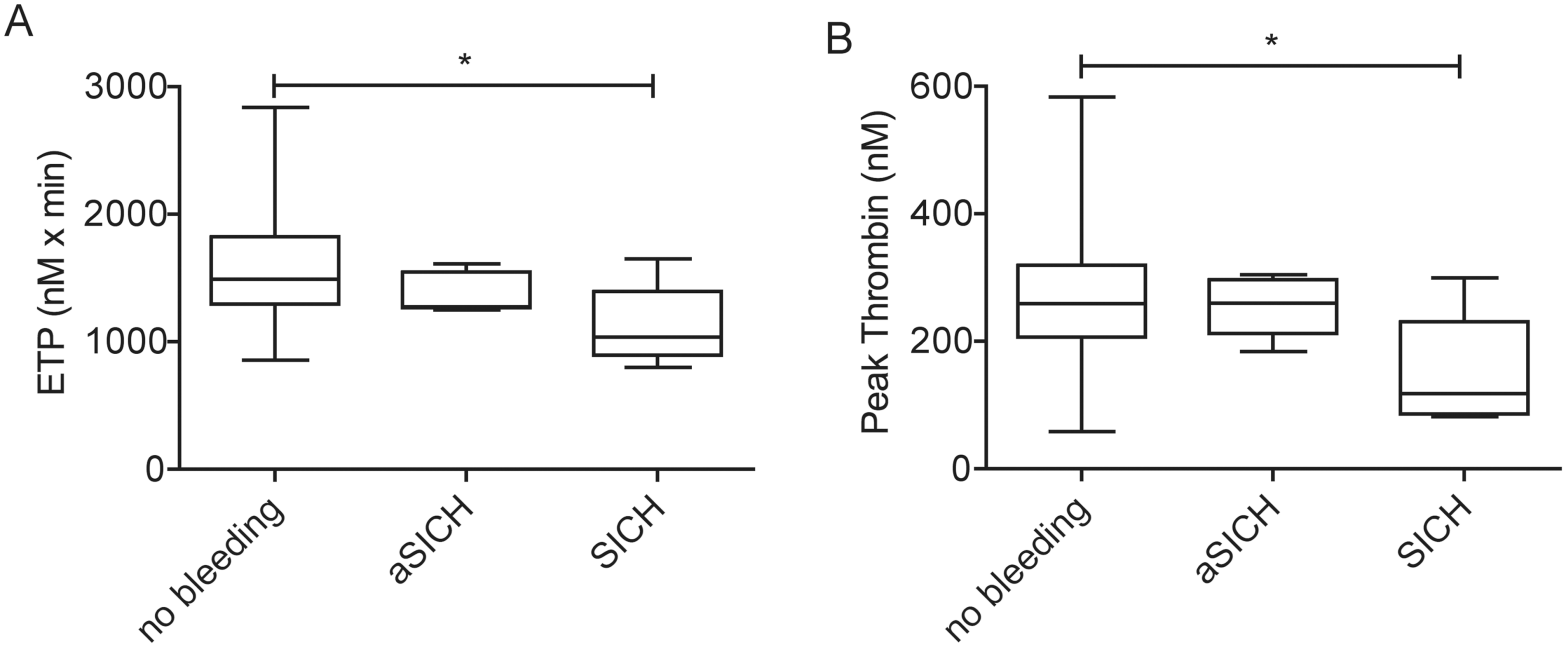
The association of (A) Endogenous Thrombin Potential (ETP) and (B) Peak Thrombin levels with therapy-associated intracranial hemorrhage. Box and whisker plots indicate median, interquartile range and total range. Statistical significance was assessed by Kruskal-Wallis and Dunn’s Multiple Comparison Test. *Statistically significant (p<0.05). ETP: Endogenous Thrombin Potential, aSICH: asymptomatic intracranial haemorrhage, SICH: symptomatic intracranial haemorrhage.

The median and IQR results of the thrombin generation parameters for the whole patient population are shown in Table 1. Among the baseline characteristics of the patient population, correlation and univariate analysis revealed no association with any thrombin generation parameters except for a weak negative association between age and the ETP and Time to Peak parameters (Spearman r = -0.25, p = 0.006 and r = -0.20, p = 0.02, respectively). No association was found between the results of any parameters of the thrombin generation test and the ASPECT scores on admission/24 hours after therapy and the time between symptom onset to blood sampling (data not shown). ETP and Peak Thrombin parameters were significantly lower in case of cardioembolic stroke as compared to that of atherothrombotic origin (Fig 1A). Other thrombin generation parameters were not associated with the subtype of stroke. Time to Peak was significantly shorter in case of more severe stroke indicating that peak thrombin generation occurs faster in these patients (Fig 1B). No association was found between the severity of the stroke and other thrombin generation parameters. ETP and Peak Thrombin parameters were significantly lower in those patients who suffered SICH as compared to the patients with no recorded bleeding complications or with aSICH (Fig 2). A multiple logistic regression analysis including age, sex, CRP, smoking, antiplatelet drug use and NIHSS on admission revealed that an ETP result under the lowest quartile (<1265.9 nM x min) significantly increased the risk of therapy-associated SICH (OR: 17.54, 95%CI: 1.45–212.72, p<0.05). Similarly, a Peak Thrombin result under the lowest quartile (<204.7 nM) on admission was an independent predictor of therapy-associated SICH (OR: 15.12, 95%CI: 1.38–166.02, p<0.05).

Low ETP and Peak Thrombin parameters were significantly associated with short-term mortality, while none of the thrombin generation parameters showed significant differences in other categories of short-term functional outcomes (favourable, no-change, unfavourable) (data not shown). A significantly lower ETP but not Peak Thrombin result was found in those patients who died by the end of the 3^rd^ month (mRS = 6) as compared to those with better outcomes (Table 3). On the other hand, as compared to patients with better functional outcomes at 3 months (mRS 0–1), ETP was not significantly lower in those patients who had worse functional outcomes (mRS 2–5) but survived by the end of the 3^rd^ month (Table 3). In a stepwise backwards regression analysis (performed with age, sex, CRP, smoking, antiplatelet drug use and NIHSS on admission) only age, lowest quartile ETP and lowest quartile Peak Thrombin results remained in the model as independent risk factors for mortality by day 14 (Table 4). Using the same model to test potential risk factors independently associated with mortality by the end of the 3^rd^ month, an ETP result in the lowest quartile, age>79 years and NIHSS>15 were found to be significant predictors, revealing odds ratios of 5.28 (95%CI:1.27–21.86, p = 0.022), 8.96 (95%CI:1.75–45.93, p = 0.009) and 5.93 (95%CI:1.31–26.80, p = 0.021), respectively (Table 4).

**Table 3:** Characteristics of the patients on admission according to clinical outcome by the end of the 3^rd^ month.

Variable	mRS 0–1 (n = 42)	mRS 2–5 (n = 30)	mRS 6 (n = 26)
Age, years	68.0 (58.0–79.0)	66.0 (58.0–77.0)	79.5 (70.0–85.0)*#†
Male	29 (69.1)	15 (50.0)	14 (53.9)
Arterial hypertension	33 (78.6)	23 (76.7)	19 (73.1)
Atrial fibrillation	11 (26.2)	4 (13.3)	9 (34.6)
Hyperlipidaemia	27 (64.3)	21 (70.0)	12 (46.2)
Diabetes mellitus	9 (21.4)	12 (40.0)	9 (34.6)
Previous stroke	12 (28.6)	12 (41.4)	7 (28.0)
Active smoker	6 (16.2)	12 (41.4)*	6 (26.1)
Serum glucose (mmol/L)	6.5 (6.0–7.6)	6.4 (5.6–8.1)	6.3 (5.5–7.5)
C-reactive protein (mg/L)	1.8 (0.8–3.6)	4.1 (1.7–5.8)*	4.4 (1.9–6.6)*†
Antihypertensive therapy	27 (73.0)	22 (75.9)	15 (62.5)
Antiplatelet therapy	18 (43.9)	14 (46.7)	14 (56.0)
Lagtime (min)	3.7 (2.8–4.3)	3.5 (2.8–4.0)	3.3 (3.2–3.8)
ETP (nM x min)	1472.6 (1291.9–1746.0)	1512.8 (1319.0–1797.1)	1275.1 (1135.3–1552.0)*#†
Peak Thrombin (nM)	268.5 ± 88.9	277.2 ± 90.7	226.8 ± 75.0
Time to Peak (min)	7.3 (6.3–8.3)	6.7 (5.7–7.7)	6.8 (6.2–8.5)
Start Tail (min)	23.4 (21.5–25.3)	23.3 (21.8–25.3)	22.9 (21.5–25.0)
Velocity Index (nM/min)	71.5 (50.6–97.2)	86.9 (60.1–140.3)	71.0 (46.2–84.3)
Time to treatment (min) with rt-PA	169.0 (120.5–181.0)	148.5 (125.0–180.0)	152.0 (130.0–176.0)
Cardioembolic stroke	9 (29.0)	5 (20.8)	6 (31.6)
NIHSS on admission	5.0 (4.0–9.0)	9.0 (6.0–15.0)*	16.0 (8.0–21.0)*†
ASPECTS on admission	10.0 (9.0–10.0)	9.5 (9.0–10.0)	10.0 (9.0–10.0)

Values are given as mean ± SD or median (IQR) or number (percentage). mRS: modified Rankin Scale, ETP: Endogenous Thrombin Potential, rt-PA: recombinant tissue type plasminogen activator, NIHSS: National Institutes of Health Stroke Scale, ASPECTS: Alberta Stroke Program Early CT Score.

*p<0.05 as compared to mRS 0–1,

^#^p<0.05 as compared to mRS 2–5,

^†^p<0.05 for all-group comparison (ANOVA or Kruskal-Wallis).

**Table 4:** Multiple logistic regression analysis for mortality.

	Odds ratio	95% Confidence Interval	*P* value
Mortality by day 14			
ETP under 1265.9 nM x min*	6.03	1.20–30.16	0.029
Peak Thrombin under 204.7 nM*	6.81	1.09–42.65	0.040
Age>79 years	6.66	1.12–39.51	0.037
Mortality by the end of the 3^rd^ month			
ETP under 1265.9 nM x min*	5.28	1.27–21.86	0.022
Age>79 years	8.96	1.75–45.93	0.009
NIHSS >15	5.93	1.31–26.80	0.021

Backward multiple logistic regression model included age, sex, CRP, smoking, antiplatelet drug use and NIHSS on admission. NIHSS: National Institute of Health Stroke Scale, ETP: Endogenous Thrombin Potential.

*Under the lowest quartile.

## Discussion

In this single-center study of 120 AIS patients treated with i.v. t-PA, we show for the first time that results of the thrombin generation test obtained before the thrombolytic treatment could be a useful marker to predict short- and long-term outcomes. Thrombin is a major effector of the coagulation process and it is an important regulator of thrombus growth and structure. In our study, a decrease in the total amount of thrombin generated in the patients’ plasma was found to be an independent predictor of mortality. In a multiple logistic regression analysis for mortality, an ETP result under the lowest quartile showed an OR: 6.03 (95%CI: 1.2–30.15, p<0.05) and OR: 5.28 (95%CI: 1.27–21.86, p<0.05) for mortality by the end of the 2^nd^ week and by the end of the 3^rd^ month after the event, respectively. The relatively low potential of thrombin generation in these plasma samples most likely reflects an intense prior or ongoing activation of coagulation leading to a consumption of coagulation factors which are normally required for thrombin generation to take place. This is in line with previous findings that in patients with coronary heart disease, a trend of lower ETP/Peak Thrombin was found in those with recurrent thrombotic events [13]. The immense activation of coagulation that is present in the first hours of AIS might not be easily traced in its complexity by other methods. In fact, the NIHSS, which is commonly used to assess the severity of the stroke, might not reflect the size and the structural features of the forming thrombus, as the symptoms of the patient are largely dependent on the location of the blood clot. In line with this hypothesis, no association was found between the NIHSS of the patients on admission and the total or maximal amount of thrombin (ETP or Peak Thrombin parameters) generated in their plasma samples. Only Time to Peak was significantly shorter in case of more severe stroke indicating that peak thrombin generation occurs faster in these patients. Our results suggest that in a fraction of the patient population eligible for thrombolysis treatment, the risk for mortality is higher, and it is associated with low thrombin generation potential. The TG method was able to differentiate between patients at high risk and low risk for mortality, however, we found no differences in the TGT results between those who survived with different outcomes as classified based on the changes in the NIHSS or the mRS score.

We also show here that besides the increased risk for mortality due to ongoing coagulation, therapy-associated bleeding risk is also higher in the patients with low TG potential. A multiple logistic regression analysis including age, sex, CRP, smoking, antiplatelet drug use and NIHSS on admission revealed that an ETP or a Peak Thrombin in the lowest quartile are independent predictors of therapy-associated SICH (OR: 17.54, 95%CI: 1.45–212.72, p<0.05 and OR: 15.12, 95%CI: 1.38–166.02, p<0.05, respectively). The prior over-activation of coagulation and the resulting decrease of the thrombin generation potential, possibly due to consumption, might result in the therapy-associated bleeding tendency in these patients. In an earlier study including 114 AIS patients undergoing i.v. thrombolysis, baseline parameters of selected coagulation and fibrinolysis markers did not yield to any significant association with SICH [14]. This result suggests the advantage of TGT over single factor determinations when testing the levels of coagulation markers as predictors of bleeding. TGT as a global test incorporates the multiple procoagulant and anticoagulant pathways in its final result, therefore it more likely represents the situation occurring in the in vivo environment. Recently, TGT has been used to predict bleeding tendency in various clinical conditions. In hemophiliac patients and in hemophiliacs with inhibitors, its usefulness has been proved, and it has been a matter of debate whether the TGT might describe the risk of bleeding better than traditional tests [15–17]. TGT, when performed preoperatively, was found to be useful in providing information predictive for blood loss after cardiac surgery [18].

Among the baseline characteristics of our patient population no association was found with any parameters of the TGT except for a weak negative association between ETP and Time to Peak parameter with age. Similar association between the Time to Peak parameter and age has been described in healthy individuals [19], and lower mean ETP results were obtained in individuals >75 years of age in a large cohort study including elderly subjects [20]. In our study significantly lower ETP and Peak Thrombin parameters were found in case of cardioembolic stroke vs. that of atherothrombotic origin. This result at first seems to contradict the results published by Rooth et al [21], who found similar thrombin generation capacity in patients with non-cardioembolic and cardioembolic strokes. However, in that study blood sampling and thrombin generation was performed within the first two weeks after the stroke event, while in our case all patients were sampled within 4.5 hours after the first symptoms of stroke. Our results are in line with those published by Gissel et al, who found that using a mathematical model to calculate thrombin generation (using plasma levels of clotting factors measured from patients sampled within 24 hours of symptom onset), all stroke sub-types except for cardioembolic had increased total thrombin produced [22]. Atherothrombotic strokes have different hemostatic risk factors than those of cardioembolic origin: it has been shown that the hemostatic profile is different and is more likely to be thrombogenic in atherothrombotic strokes as compared to cardioembolic strokes, which might explain our findings [23].

Our study has some limitations. First, the number of patients with symptomatic hemorrhage was small, which was obviously due to the fact that it was a single-site study with a limited number of eligible patients per year. However, as thrombin generation measurements are highly sensitive to pre-analytical variables, including the method of blood collection, plasma isolation and sample storage conditions [24], up to the point where these variables may significantly influence assay results [25], the study was designed to be single-centered in order to assure unified sample handling. As results were significantly different in case of patients with or without SICH, the possibility of a type II error should not be considered in our case. Second, in theory, some patients might have had inherited thrombophilia risk factors influencing TG results and this was not tested. However, as the youngest patient having stroke in this cohort was 59 years old and all patients had negative history for venous thromboembolism, the occurrence of major thrombophilia risk factors influencing the results seemed rather low in this cohort and did not seem worthwhile to perform such an exhaustive test panel to rule out this possibility. Third, thrombin generation test has a number of limitations. The assay is still not standardized enough for broad clinical use and there is a lack of reference range for specific conditions, including the condition we have used in this study [26]. It was not our aim in this study to develop a reference range tested on healthy individuals and therefore no such population was enrolled here. Although standardization of the TGT is far from being completed, optimal experimental conditions have been described previously, which were followed by us in this study [26, 27]. This includes, among others, the use of a standardized thrombin calibrator, which allows the quantitative expression of thrombin, the use of the Thrombogram software, which can reliably describe the distinct phases of thrombin generation, and the practice that all samples were run in duplicates.

## Conclusions

We have shown for the first time that the TGT might be a useful tool to predict outcomes in AIS patients undergoing thrombolysis. Patients with low thrombin generation potential (low ETP parameter) on admission were found to have an increased risk of worse therapeutic outcomes including therapy-associated symptomatic intracranial bleeding or death. Prospective studies are needed in the future in order to test whether these selected patients might benefit from altered and/or supplemented therapeutic profiles.
